# Electrophysiological Evidence for Spatiotemporal Flexibility in the Ventrolateral Attention Network

**DOI:** 10.1371/journal.pone.0024436

**Published:** 2011-09-12

**Authors:** Jelena Ristic, Barry Giesbrecht

**Affiliations:** 1 Department of Psychology, McGill University, Montreal, Canada; 2 Department of Psychological and Brain Sciences and the Institute for Collaborative Biotechnologies, University of California Santa Barbara, Santa Barbara, California, United States of America; Royal Holloway, University of London, United Kingdom

## Abstract

Successful completion of many everyday tasks depends on interactions between voluntary attention, which acts to maintain current goals, and reflexive attention, which enables responding to unexpected events by interrupting the current focus of attention. Past studies, which have mostly examined each attentional mechanism in isolation, indicate that volitional and reflexive orienting depend on two functionally specialized cortical networks in the human brain. Here we investigated how the interplay between these two cortical networks affects sensory processing and the resulting overt behavior. By combining measurements of human performance and electrocortical recordings with a novel analytical technique for estimating spatiotemporal activity in the human cortex, we found that the subregions that comprise the reflexive ventrolateral attention network dissociate both spatially and temporally as a function of the nature of the sensory information and current task demands. Moreover, we found that together with the magnitude of the early sensory gain, the spatiotemporal neural dynamics accounted for the high amount of the variance in the behavioral data. Collectively these data support the conclusion that the ventrolateral attention network is recruited flexibly to support complex behaviors.

## Introduction

Coherent behavior critically depends on interacting attentional mechanisms that maintain one's current attentional focus (e.g., looking left and right before crossing the street via the mechanism of volitional orienting) and facilitate reorienting of attention to behaviorally relevant events in the environment (e.g., monitoring for other pedestrians and parked cars via the mechanism of reflexive orienting). While a large number of studies investigated the cognitive and neural mechanisms of volitional and reflexive systems independently (e.g., [1]), comparatively less is known about how the two systems interact to produce complex behaviors. We investigated this issue by delineating the temporal and spatial characteristics of neural and behavioral responses when participants perform a task, which, like most typical everyday activities, requires engagement of both volitional and reflexive attentional control mechanisms.

The control of human attention in the brain, both volitional and reflexive, depends on the dynamic interplay between multiple brain regions. According to one proposal [1], the key regions form two distinct networks: a *dorsolateral frontoparietal network*, which includes parietal cortex (superior parietal lobe, SPL; intraparietal sulcus, IPS) and the frontal eye fields (FEF) bilaterally [2], [3], [4]; and a *ventrolateral frontoparietal network*, which includes the temporal-parietal junction (TPJ), middle frontal gyrus (MFG), and ventral frontal cortex (VFC, including the inferior frontal gyrus, IFG) in the right hemisphere. A number of studies have demonstrated dissociations between these networks as a function of the manner in which attentional orienting is triggered. The dorsolateral network controls the current attentional focus independent of whether orienting is triggered volitionally by internal goals or whether it is triggered in a more reflexive, stimulus-driven fashion [5]. The ventrolateral network, on the other hand, tracks behaviorally relevant contingencies [6], [7], [8], responds to violations of expectation [9], and initiates reorienting to salient events via MFG, which acts as a circuit-breaker by disrupting the current focus of attention maintained by the dorsolateral network (e.g., [5]).

Recent work has shown that not only is the ventrolateral network functionally distinct from the dorsolateral network, but also that the regions within the ventrolateral network display functional specificity. Posterior regions (i.e., TPJ) respond to frequent violations of expectation, typically observed during reflexive reorienting whereas anterior regions (i.e., IFG) respond to infrequent violations of expectation, typically observed during volitional reorienting [9]. This framework for understanding the reorienting of attention to unexpected events makes two predictions. First, due to their functional specificity, posterior and anterior regions of the ventrolateral network should simultaneously index violations of different types of expectancies. Second, if MFG acts as a circuit-breaker, attentional reorienting elicited by events that generate automatic contingencies should be marked by initial activity in the MFG.

To examine these hypotheses, we recorded participants' EEG while they performed a spatial cuing task that induced both an explicit contingency, engaging volitional orienting, and an automatic contingency, engaging reflexive orienting. We generated the explicit contingency by associating the numerically small cue (3) with a target occurring on the right and the numerically large cue (9) with a target occurring on the left. Importantly, the use of number cues in this task also generated a concurrent automatic contingency because perception of numerically small numbers reflexively shifts attention to the left side of space and perception of numerically large numbers reflexively shifts attention to the right side of space [10], [11]. The neural responses of the ventrolateral regions in this experimental condition were compared to a control condition in which central number cues did not predict the location of the target and thus induced only an automatic contingency. To understand the dynamics of the attentional mechanisms when both volitional and reflexive processes are engaged, we examined the spatio-temporal characteristics of target-related EEG activity using a multiple source beamformer analysis (MSBF; [12], [13]), and related it, using multiple linear regression, to visual responses evoked by the target, measured using a traditional event-related potential (ERP) approach, and behavioral performance.

## Results

We conducted two types of analyses to test our hypotheses. The first examined behavioral performance and ERP responses evoked by the target stimuli. The second examined the spatial and temporal dynamics of the ventrolateral regions using the MSBF technique.

### RT and ERP Results

We focused our analyses on the longest cue-target interval for three reasons. First, by allowing ample time between the presentations of the cue and the target, we alleviated any concerns about potential differential overlap between the neural activity created by temporally adjacent events (e.g., [14]). Second, based on previous studies showing diminished temporally early effects during cued attention tasks with explicit contingencies (e.g., [15], [16], [17]), we did not expect behavioral effects in the experimental condition to emerge prior to 600 ms after cue presentation. Third, based on past research showing that the mental number line does not affect automatic orienting prior to 600 ms after cue onset [10], [11], [18], [19], we also did not expect to observe behavioral facilitation in the control condition prior to this cue-target time interval.

Confirming these predictions and replicating past data showing facilitated performance for congruent targets when the task manipulates explicit contingencies (e.g., [15]), but not automatic contingencies elicited by a mental number line (e.g., [11], [20]), congruent targets were detected faster than incongruent targets. This was verified using a within subjects ANOVA with condition (experimental, control) and target congruency (congruent, incongruent) included as factors. This analysis returned a significant interaction between condition and target congruency [F (1,8) = 8.94, p<0.02] indicating that, in contrast to the control condition, congruent targets in the experimental condition were detected faster than incongruent targets. Mean interparticipant RTs and P1 amplitudes for the experimental and control conditions as a function of cue-target congruency and SOA are shown in Table 1.

**Table 1:** Interparticipant mean RTs, P1 amplitudes, and standard deviations as a function of cue-target congruency for the experimental and control conditions.

Interparticipant Means	CONDITION											
	Experimental						Control					
	RT		P1 Contralate		P1 Ipsilateral		RT		P1 Contralater		P1 Ipsilateral	
	*M*	*SD*	*M*	*SD*	*M*	*SD*	*M*	*SD*	*M*	*SD*	*M*	*SD*
100 ms SOA												
* Congruent*	331	27	5	2.5	5.7	2.3	340	35	5.3	3.1	5.7	3.6
* Incongruent*	334	31	4.9	2.8	5.2	2.7	339	36	4.9	3.3	5.2	3.1
300msSOA												
* Congruent*	280	24	-.4	1.7	-.1	1.7	292	25	.7	2.5	.7	2.7
* Incongruent*	289	23	-.2	1.8	-.1	1.3	293	25	.5	2	.8	2.3
600ms SOA												
* Congruent*	271	29	2.6	.6	2.7	1.5	290	34	3.4	1.4	4.3	1.7
* Incongruent*	286	34	2.0	.7	2.8	1.6	287	34	2.5	1	3.8	2.0

**Figure 1:**
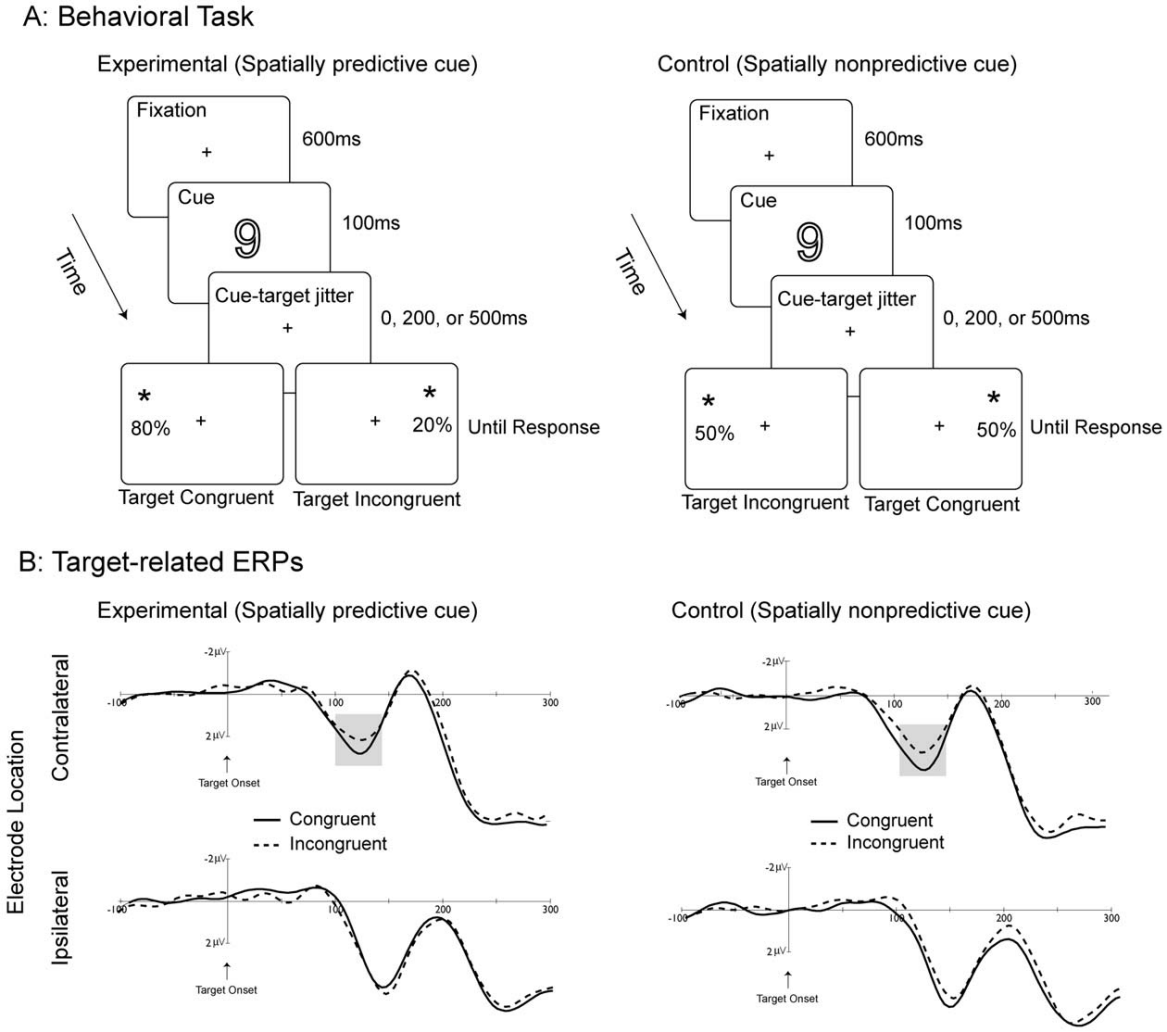
Stimuli and Results. **1A**: Example sequence of trial events and illustration of stimuli for the experimental and control conditions. Fixation display in duration of 600 ms was followed by a presentation of the central number cue (3 or 9) in duration of 100 ms. The target (occurring in either left or right upper visual field) demanding a detection response appeared after 0, 200 or 500 ms. The stimuli are not drawn to scale. **1B**: Target-related ERPs. Target related modulation of P1 amplitude for experimental and control conditions as a function of electrode site and cue-target congruency averaged across posterior occipital electrodes P5, P7, PO7, and PO3 on the left, and P6, P8, PO8, and PO4 on the right.

If explicit and automatic contingencies influenced attentional orienting, one would expect to observe changes in P1 amplitude as a function of the explicit contingency in the experimental condition and the automatic contingency in the control condition. Furthermore, based on prior literature (e.g., [21]), we also expected to observe larger P1 amplitudes at electrodes contralateral to the target location relative to electrodes ipsilateral to the target location. Average target-evoked ERP waveforms at contralateral and ipsilateral electrodes are shown in Figure 1b. Consistent with our predictions and the literature [21], [22], [23], the magnitude of the P1 was larger for congruent relative to incongruent targets across contralateral compared to ipsilateral electrode sites. This observation was confirmed by a repeated measures ANOVA with condition (experimental, control), electrode location (ipsilateral to the target, contralateral to the target), and target congruency (congruent, incongruent), which revealed a significant two-way interaction between electrode location and target congruency [F (1,8) = 9.16, p<0.02]. This critical interaction demonstrates that (1) larger P1 amplitudes were observed for the electrode sites contralateral to the location of the target, indicating that the attention shifts elicited by both experimental and control conditions modulated early visual processing of the target; and (2) P1 magnitude was modulated by the explicit contingency in the experimental condition and the left-to-right mental number line automatic contingency in the control condition. That is, in the experimental condition the amplitude of the P1 was enhanced for the targets occurring on the on the left appearing after a numerically large cue (9), and targets occurring on the right appearing after a numerically small cue (3). In contrast, in the control condition, the amplitude of the P1 was enhanced for the targets occurring on the left appearing after a numerically small cue (3), and targets occurring on the right appearing after a numerically large cue (9). The data from the control condition provide an important verification that central digit cues generated automatic contingencies when they were not informative of the target location, thus emerging *before* behavioral effects are typically observed. A main effect of condition [F (1,8) = 15.4, p<0.01], indicating overall more positive voltage for the control condition, and an interaction between condition and electrode location [F (1,8) =  9.12, p<0.02], indicating overall more positive voltage for ipsilateral compared to contralateral electrode sites for the control compared to experimental condition were also reliable.

### MSBF Results

**Figure 2:**
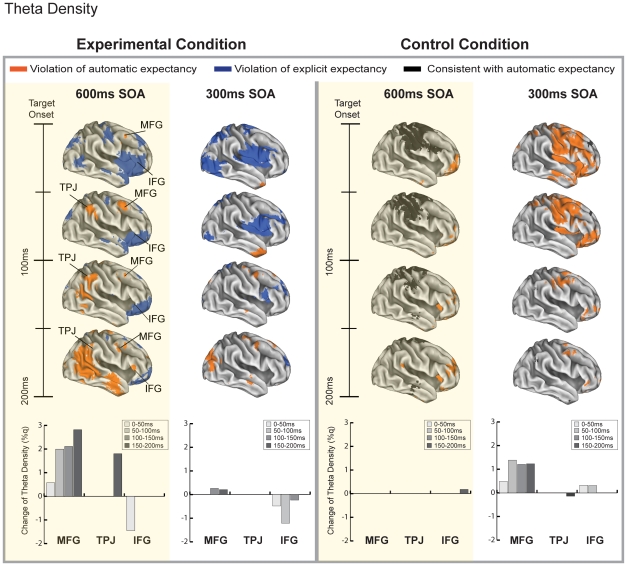
MSBF Results. Significant voxel values (p<.00001) indexing the approximate locations of cortical regions showing modulations of theta density in a direct contrast between congruent target (violations of automatic expectancy, depicted in orange) and incongruent target (violations of explicit expectancy, depicted in blue) occurring before response execution in the experimental and control conditions at 600 and 300 ms SOA. Bar graphs depict the significant theta density change for each ventrolateral region of interest (MFG [x = 45, y = 6, z = 46], TPJ [x = 52, y = −50, z = 28], and IFG [x = 48, y = 14, z = 10]) as a function of time.

The multiple source beamformer analysis examined changes in theta density in regions of the ventrolateral attention network to test the hypotheses that (a) posterior and anterior regions of the ventrolateral network are able to simultaneously index violations of different types of expectancies, and (b) if MFG acts as a circuit-breaker, then conditions in which attentional reorienting elicited by events that generate automatic contingencies should be marked by initial activity in the MFG. We analyzed the theta frequency band because previous work has shown that this is a potential critical carrier frequency for long range communication between cortical regions involved in attentional control [12]. Figure 2 illustrates the results of the MSBF analysis for the experimental and control conditions. The images in Figure 2 depict voxels showing statistically significant change of theta density (%q, computed relative to pre-target baseline, see Methods) as revealed by a direct contrast between the trials in which violations of automatic expectations occurred (i.e., congruent target) and the trials in which violations of explicit expectations occurred (i.e., incongruent target) shown for each condition and SOA separately. The bar graphs show the mean change in theta density within each ventrolateral subregion of interest (i.e., MFG, TPJ, and IFG) as a function of time.

#### Experimental Condition

The MSBF analysis of the experimental condition, in which both volitional and automatic processes were engaged, revealed three key results. First, modulations in theta density in response to violations of expectancy were observed in MFG, TPJ and IFG regions of the right lateralized ventrolateral network (see Figure 2, 600 ms SOA). The pattern of activity was marked by an early increase of activity in both MFG and IFG, followed by later activity in TPJ. Second, the activity in these subregions varied as a function of the type of expectancy violation. While MFG and TPJ responded to automatic contingency violations, IFG responded to explicit contingency violations. Third, the activity of the ventrolateral subregions varied as a function of time. The activity in MFG and IFG increased relative to baseline within the first 50 ms after target presentation; However each subregion responded to a different type of expectancy violation. The early activity in MFG was followed by the later engagement of TPJ, which started responding in concert with MFG by 100 ms post-target. MFG and TPJ continued to index violations of the automatic contingency throughout the epoch while IFG, after initial activity in response to violations of the explicit contingency, also responded to violations of the automatic contingency. Changes in theta activity were also observed within occipitotemporal regions − including extrastriate visual areas and middle temporal gyrus − as well as in the parts of the dorsolateral network − including precentral sulcus and the parietal lobe—presumably reflecting the maintenance of the explicit contingency [2], [12].

**Figure 3:**
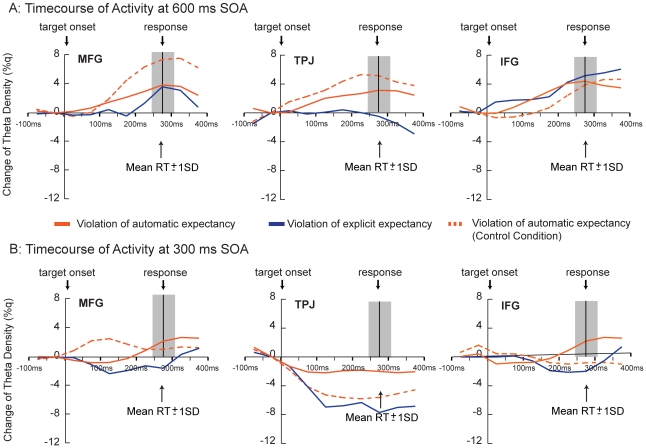
The time course of the change in theta density for 600 ms SOA (3A) and for 300 ms SOA(3B). The data show percent change in theta density (%q) for each 50 ms time interval relative to 50 ms pre-target baseline for MFG [x = 45, y = 6, z = 46], TPJ [x = 52, y = −50, z = 28], and IFG [x = 48, y = 14, z = 10] ROIs for the experimental and control conditions as a function of target congruency. The data reflect the percent theta change computed for each 50 ms time bin across the entire trial period (including the cue activity, target activity, and 100 ms of post target activity) relative to 50 ms pre-cue baseline. Then, for each time bin, these theta density values were normalized to the 50 ms pre-target activity. The shaded area depicts ±1SD of the overall mean of the response in the experimental condition (278 ms). Please note that the time resolution of the MSBF analysis is limited to 50 ms.

The time courses of the average change in theta density (relative to baseline) for each ventrolateral subregion of interest as a function of condition, target congruency, and time bin are shown in Figure 3A. The data dovetail with those from the whole-brain MSBF contrasts and indicate that while MFG and TPJ showed increased theta density in response to violations of automatic expectancy, IFG showed increased theta density in response to violations of explicit expectancy. Finally, these time courses also show that the increased theta density observed in the IFG, which has been implicated in the control of response conflict [24], appears to be driven by the response conflict underlying the cue-target congruency rather than response execution itself, as the activity in the IFG diminishes after response.

#### Control Condition

If the unique temporal dynamics of the ventrolateral regions is determined by the conditions in which the two attentional networks compete for resources, one could predict that these dynamics should change when only one type of contingency is present. The data from the control condition support this notion (see Figures 2 and 3A). First, when only an automatic contingency was present in the control condition, the ventrolateral subregions responded with a different spatiotemporal profile. Replicating previous studies indicating a critical role of TPJ in reorienting of attention [1], Figure 3A shows an increase in theta density for incongruent targets in TPJ. In contrast to the experimental condition, this response occurred later, at 200 ms post-target. Further, activity in MFG and IFG was not observed. The absence of the early activity in MFG suggests that reorienting signals were not needed because the dorsolateral network was engaged by a single task. The comparatively lower response of the MFG relative to the experimental condition also suggests the possible suppression of MFG activity when only single contingency is present in the environment. The absence of early activity in IFG is consistent with the notion that the control task did not generate explicit contingencies.

Finally, Figure 2 also shows the data for the trials in which the target location was congruent with the automatic expectancy created by the mental number line. Here we observed activity in the dorsolateral network, including parts of the superior parietal lobe, central sulcus, pre-central sulcus, and FEF within 150 ms post-target. This time course of activity is consistent with the timing of the P1, and suggests that the modulation of the P1 observed in the control condition was related to activity in the dorsolateral network. This result is consistent with past reports showing that P1 can be modulated by both voluntary [22] and reflexive processes [25] and once again, dovetails with the recent notion that the dorsolateral network appears to be responsible for controlling attentional orienting under conditions when there is no competition between volitional and reflexive processes [5].

Together, the results of the MSBF analyses support the conclusion that functionally specialized regions of the ventrolateral network track violations of automatic and explicit expectancies only when both types of contingencies are present in the environment and when the two attentional systems interact. Within this context, the activity of the subregions is marked by specific temporal dynamics with early MFG and IFG changes in theta density followed by the later response modulation of TPJ. When only one type of contingency is present in the environment, the dorsolateral network appears to maintain responses to both explicit and automatic contingencies.

### Regression Results

**Figure 4:**
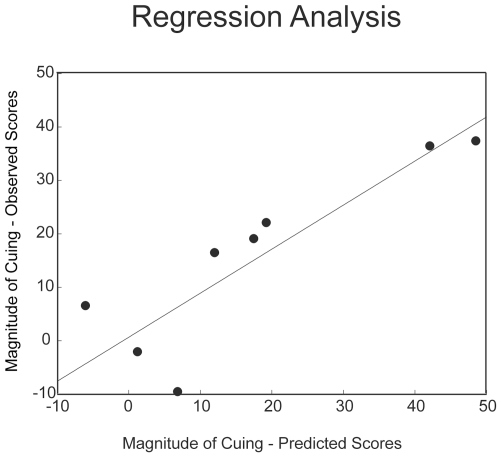
Regression Results. The relationship between the individual participants' predicted score in the experimental condition using the unstandardized coefficients obtained by the regression analysis (mean theta density in MFG and IFG between 0–50 ms; mean theta density in TPJ between 50–100 ms; the magnitude of the P1 for contralateral electrode sites; the magnitude of the P1 for ipsilateral electrode sites) on the x-axis and the magnitude of the individual participants' behavioral effect on the y-axis (R^2^ = 0.97).

The underlying assumption in neuroimaging studies is that the changes in brain responses are critically involved in supporting behavior. In the present context, this assumption predicts that the activity in the ventrolateral network, along with the ERP results, should account for a significant portion of the variability in the RT data in the experimental condition. We tested this hypothesis using a multiple linear regression analysis that included the individual mean theta density for each ROI (as revealed by the direct contrast in the experimental condition) and the magnitude of both the contralateral and the ipsilateral P1 (Congruent P1-Incongruent P1) as predictors of the magnitude of each individual's behavioral cuing effect (Congruent RT- Incongruent RT). To characterize the importance of the observed temporal dynamics (i.e., the early and simultaneous response of MFG and IFG), we included MFG and IFG activity between 0 and 50 ms and TPJ activity between 50 and 100 ms. Figure 4 shows the scatterplot of the predicted and the measured values along with the estimated linear fit. Consistent with our hypotheses, this model accounted for 97% of the variance in the RT data (R2 = 0.97, F (3,8) = 18.09, p<0.02). Although this regression model was based on a relatively small sample and therefore must be interpreted with caution, control models with the same number of parameters, which included ROI activity with temporal parameters that do not conform to the hypothesized model (Model 1 =  MFG, IFG and TPJ from 0–50 ms; Model 2 =  TPJ, IFG from 0–50 ms, MFG from 50–100 ms; Model 3 =  TPJ from the 0–50 ms and IFG and MFG from 50–100 ms) failed to reach statistical significance (all Fs<4.5, all ps>0.12). In addition to demonstrating that a specific spatiotemporal profile of responses in subregions of the ventrolateral network is predictive of behavior, these control analyses also indicate that the high R2 observed in the main model is likely not due to the number of predictors included in the model. Thus, the results of the regression analysis indicate that behavioral outcomes are fundamentally related to the spatial *and* temporal dynamics of the activity in the ventrolateral subregions.

### Analyses of the Short Cue-Target SOAs

While there are strong theoretical and methodological reasons for focusing our main analyses on the 600 ms SOA condition, we also examined performance and theta density for the short SOA condition. If the spatiotemproal dynamics observed at the 600 ms SOA is unique to the case when both reflexive and volitional control systems are engaged, a different pattern of results is expected in the conditions when these mechanisms are not engaged, i.e., at earlier SOAs. Overall, the analyses of the short SOA data support this hypothesis.

RT performance at SOAs of 100 and 300 ms was examined in a within subjects ANOVA with SOA (100, 300 ms), condition, and cue-target congruency included as factors. This analysis returned no reliable effects or interactions involving condition or cue-target congruency (all Fs<4, all ps>.08). The only significant effect was a main effect of SOA [F (1,8) = 95.8, p< .0001] indicating a standard foreperiod effect where overall RTs become shorter with increasing SOA (e.g., [26]). Similarly, no modulations in P1 magnitude as a function of condition or target congruency were observed. This was confirmed by a within-subjects ANOVA conducted on early SOAs (100 ms, 300 ms), condition (experimental, control), electrode location (contralateral, ipsilateral), and target congruency (congruent, incongruent). This analysis returned no effects or interactions involving condition or target congruency (all Fs <3.5, all ps> .1). The only reliable effects were a main effect of SOA [F (1,8) = 14, p<.01], indicating overall more positive voltage for the earliest SOA of 100 ms, and a main effect of condition [F(1,8) = 6.5, p<.05], indicating overall more positive voltage for the experimental condition (see Table 1).

Dovetailing with these data, the MSBF results also indicated a markedly different spatiotemporal profile of activity relative to the 600 ms SOA condition. As shown in Figure 2, at 300 ms SOA in the experimental condition, virtually no significant differences in the ventrolateral regions were observed, save for the early transient IFG response in response to the violations of explicit contingencies. Reliable changes in theta activity were observed in the occipitotemporal regions and in the parts of the dorsolateral network, which, like in the 600 ms SOA analysis, likely reflect the maintenance of the task set [12]. In the control condition, MFG showed increased theta density throughout the entire epoch in response to automatic contingency violations while IFG transiently indexed those violations within the first 100 ms post target. While this pattern of activity appears similar to the data in the 600 ms experimental condition, it is important to note two key differences. One, there was no TPJ activity. This is consistent with the notion that automatic contingencies were not tracked and dovetails with the present finding indicating diminished behavioral and early visual processing effects in this condition. Two, the magnitude of the MFG response was once again comparatively lower than the magnitude of the MFG response in the 600 ms experimental condition, thus potentially failing to activate TPJ and initiate reorienting response. Finally, a clear dissociation between the long and short SOA conditions is demonstrated in Figure 3B which shows the time course of theta density for the 300 ms SOA as a function of cue-target congruency and time bin. Importantly, and in contrast to the critical 600 ms condition, there were negligible increases in theta density relative to baseline were observed across the three ventrolateral subregions of interest.

## Discussion

We investigated the relationship between the spatial and temporal activity in the ventrolateral attention network when both volitional and reflexive attention are engaged by a single task. Recent studies demonstrate that instead of being functionally homogenous, subregions of the ventrolateral network are functionally dissociable [9]. The posterior parts (i.e., TPJ) are engaged during reflexive reorienting while the ventral anterior parts (i.e., IFG) are engaged during volitional reorienting [9]. In this framework, MFG [5] is hypothesized to play a key role in initiating reflexive reorienting, as it acts to disrupt ongoing activity of the dorsolateral network. We examined whether the posterior and the anterior parts of the ventrolateral network can be engaged in parallel by violations of explicit and automatic contingencies. We also examined whether reflexive reorienting is initiated by the activity in the MFG. We measured the spatio-temporal dynamics of activity in the ventrolateral regions (i.e., MFG, TPJ, and IFG) using a novel electrical neuroimaging technique [12] and related it to visual processing of the target and behavioral performance.

Our results support both hypotheses. Verifying the effectiveness of our experimental task, behavioral and ERP results demonstrated that attention was modulated by task-relevant explicit contingencies. We observed facilitation in RTs and an associated increase in P1 amplitude for the targets occurring at attended locations. The results of the MSBF analysis indicated that right lateralized MFG and TPJ responded to violations of automatic contingencies while right IFG responded to violations of explicit contingencies. Importantly, the activity in different ventrolateral regions was marked by a specific time course of activation. Consistent with the idea that different subregions of the ventrolateral network concurrently track different types of environmental contingencies, with MFG indexing violations of automatic expectancy and IFG indexing violations of explicit contingency, showed an increase in theta density within the first 50 ms after target onset. Supporting the idea that reflexive reorienting may critically depend on the initial activity in MFG, the early increase in theta density in MFG was followed by the later increase in theta density in TPJ. Importantly, this MFG activity was absent when the task did not induce competition between volitional and reflexive orienting, and thus did not require switching between the two networks. Finally, the time course of activity in MFG, TPJ, and IFG, and the magnitude of the target-related P1 in the experimental condition accounted for the significant amount of the variability in the behavioral data.

These results have three key implications. First, capitalizing on the excellent temporal resolution of EEG, we found that dorsal and ventral subregions of the ventrolateral frontal cortex respond simultaneously, within the first 50 ms, to violations of different types of contingencies. The dorsal regions (i.e., MFG) appear to be dedicated for monitoring and responding to automatic contingencies that may be present in the environment. The ventral regions (i.e., IFG) appear to be dedicated for maintaining attentional focus in accordance with the current task. Interestingly, our data also revealed that after the early response to the violations of explicit contingency, IFG also responded to violations of automatic contingency peaking very close to the response. This result may reflect the hypothesized role of the right IFG in inhibition of prepared responses [24], which in the present context includes those prepared for responding to the automatic contingencies generated by the left-to-right mental number line. This interpretation is consistent with the results of past behavioral studies, which show that reflexive orienting elicited by the mental number line is suppressed by concurrent volitional orienting [11], [18], [19]. Thus, right IFG might play a key role in mediating apropriate responses when two processes compete for attentional resources. While our data support this conclusion, future studies are required to precisely elucidate the function of the right IFG in attentional selection.

Second, the temporal progression of MFG and TPJ activity in response to violations of automatic contingencies strongly suggests that activity in TPJ typically observed during reflexive reorienting depends on the early and transient activity in MFG. This is consistent with the model of attentional control networks in which MFG is conceptualized as an important structure that relays information between the ventrolateral and the dorsolateral networks (e.g., [5]). This pattern of activity is also consistent with the recent conceptualization of the role of the ventrolateral network in detection of behaviorally relevant contingencies in the environment [5], [6]. Extending these past data, the results from our experimental and control conditions strongly suggest that the initial activity in MFG is the critical driving factor in the initiation of reflexive reorienting when the dorsolateral network is engaged by an ongoing task. To our knowledge these results are among the first to unambiguously demonstrate early and critical involvement of frontal areas in attentional control. Importantly, our data dovetail with recent studies showing early engagement of the frontal regions in the control of attentional and perceptual processes [12], [27] and solidify evidence for the important role of feedback neural connections in the control of visual attention [28].

Third, we also show that the variability in the magnitude of the behavioral effect is related to the neural dynamics of the ventrolateral network. If subjects were keeping track of the automatic mental number line in the experimental condition, it is reasonable to expect that this process would account for some of the variability in the behavioral effect. This is exactly what our regression analyses indicated. Importantly, the goodness of fit was critically determined by the temporal progression of activity in the ventrolateral regions, dovetailing with the conceptualization of activity in MFG operating as a ‘circuit-breaker’ that triggers reorienting in response to behaviorally relevant contingencies. Thus, the results of the regression analysis offer strong support to the notion that observed behavior is fundamentally related to the spatiotemporal activity in the attentional control networks.

Collectively, our data support the conclusion that the ventrolateral attention network plays a key role in mediating complex behaviors. We show that the ventrolateral subregions are recruited and respond flexibly depending on the nature of the incoming sensory input, its relation to internal expectations, and the behavioral goals required for performing the task at hand. One of the key structures mediating attentional allocation appears to be MFG, which tracks behaviorally relevant contingencies in the environment and facilitates communication between the two attentional networks [5]. Thus, when volitional and reflexive orienting mechanisms are engaged at the same time, as it is often the case in real life, adaptive behavior depends on the rapid activation of the specialized regions of the ventrolateral network. Moreover, our data also suggest that this response appears to depend on the low frequency theta oscillatory activity (e.g., 4–7 Hz; [29], [30], [31]), which has been associated with synchronization of responses of attended sensory inputs [30], [32], facilitation of communication between distant brain areas [12], and appears to be synchronized with high frequency gamma oscillations (e.g., 30–1000 Hz; [33]), which have traditionally been associated with attentional processes [34].

Finally, our findings also speak to the prevailing theoretical conceptualization of attentional control processes. Our results suggest that the distinction between voluntary and reflexive attentional control does not map directly onto separable underlying neural networks, as it was previously proposed (e.g., [1]). Instead, our results offer support for a more recent model in which attentional control is conceptualized to be mediated mostly by the structures associated with the dorsolateral network. According to this novel view, the ventrolateral network is involved in monitoring behaviorally relevant contingencies in the environment [5]. Our results extend this model and indicate that the ventrolateral regions play an active role in mediating the responses of the attentional networks to behaviorally relevant events and critically influence behavioral outcomes and sensory processing of attended events. Together these data suggest an expanded understanding of the neural correlates of human attention, their role in stimulus selection and processing, and their relationship with overt behavior, and as such represent an exciting new venue for the future studies of human attention.

## Materials and Methods

### Participants, Apparatus and Stimuli

Volunteers (n = 9, 5 male, mean age = 23) completed a spatial cuing task in which a centrally presented cue (either a 3 or a 9, each 2.75°×0.91°) was followed by a presentation of a target (an asterisk, *) in either the upper left or upper right visual field (3.4° eccentricity). All stimuli were black on a white background and presented on a 19-inch CRT display. All experimental procedures were approved by the University of California Santa Barbara Research Ethics Board. Written informed consent was obtained from all participants.

### Design and Procedure

Number magnitude (3 vs. 9), cue-stimulus jitter (100, 300, 600 ms), and target location (left, right) were manipulated within subjects and presented in random order. In the experimental condition, the target was 80% likely to occur on the right following a numerically small cue (3) and 80% likely to occur on the left following a numerically large cue (9). This design yielded two types of trials. In the *congruent* trials, the target appeared at the likely location (p = .8). This provided an index of violations of automatic expectancy, as these trials were inconsistent with the automatic left-to-right number line. In the *incongruent* trials, the target appeared at an unlikely location (p = .2). This provided an index of violations of explicit expectancy, as these trials were inconsistent with explicitly generated contingency.

To examine the response in the ventrolateral subregions when the competition between volitional and reflexive orienting is removed, the same participants completed a control task in which digit magnitude did not predict the location of the target (p = .5). Now the *incongruent* condition (e.g., a target occurring on the left following a numerically large cue) provided an index of violation of automatic expectancy while the *congruent* condition provided an indication of the effects that are consistent with the automatic expectancy created by the left-right mental number line. The order of experimental and control conditions was counterbalanced across participants.

Each trial began with a 600 ms presentation of a fixation cross − subtending 0.23°− followed by the presentation of the cue for 100 ms (see Figure 1A). After a variable time interval (0, 200, or 500 ms), a target, demanding a speeded detection response, appeared on the screen, thus producing Stimulus Onset Asynchronies (SOAs) of 100, 300, and 600 ms. The target remained on the screen until response was made or 2700 ms had elapsed, whichever came first. On 6% of trials, a target was not presented, and participants were instructed to withhold their response. The intertrial interval was a random interval between 750 and 1250 ms. All participants were instructed about cue-target contingencies, viewed the stimulus display from a distance of 125 cm, and maintained central fixation through the experiment, which was verified by EOG recordings. Participants completed of total of 2520 trials (1260 trials in each condition).

### Behavioral Data Analysis

Anticipations, defined as RTs less than 100 ms (Experimental  = 1.38%; Control = 1.34%;), timed out responses, defined as RTs above 1000 ms (Experimental =  0.09%; Control = 0.15%), and incorrect key presses (Experimental = 0.22%; Control = 0.27%) were infrequent. Similarly, false alarms on target absent trials were rare (Experimental = 5.24%; Control = 6.66%). Error rates were equal across the two conditions and between congruent and incongruent trials within each condition (all ts<0.8, all ps>0.26). All incorrect trials were removed from the data analyses and were not analyzed further. The order of condition presentation did not interact with cue validity in either RT or EEG data (all Fs<3, all ps>0.1).

### EEG Recording and Data Analysis

Electroencephalography (EEG) data were acquired using a BioSemi ActiveTwo system from 64 Ag/AgCl electrodes positioned according to the international 10–20 system and digitized at 256 Hz. Horizontal and vertical electrooculograms (EOG) were recorded from electrodes placed above and below left eye and 1 cm lateral to the left and right canthii. The data were referenced offline to the average of the left and right mastoids and bandpass filtered (.1–30 Hz). Trials that included ocular artifacts (EOG amplitudes exceeding ±50 µV) were excluded (Experimental condition = 8.1%; Control condition =  5.35%). Mean event-related potentials (ERPs) were created by time locking the data to the onset of the target. Epochs included 300 ms post-target activity computed relative to 100 ms pre-target baseline. The P1 ERP component was defined as ±10 ms of the peak positive amplitude occurring between 90–140 ms in the grand average waveform of the 600 ms SOA condition. This identified a common P1 window between 117 and 136 ms for the contralateral electrodes and between 133 and 153 ms for the ipisilateral electrodes. P1 mean amplitude for the earlier jitter intervals was extracted across the same time windows. P1 amplitude in all conditions was examined at posterior occipital electrodes P5/6, P7/8, PO7/8, PO3/4. These electrode sites show maximal positive amplitudes in the studies of attentional modulation of sensory processing and roughly correspond to the spatial locations of the estimated cortical sources of the P1 [25], [35].

### MSBF Analysis

MSBF was implemented in Brain Electrical Source Analysis software (BESA 5.1; Megis [36]). Raw EEG data were time locked to the onset of the target. Each trial included 50 ms of pre-target baseline and 300 ms of post-target activity. A complex demodulation technique was applied to each trial, across electrodes, resulting in a time-frequency evolution of the spectral magnitude density (normalized to pre-target baseline) for frequencies between 2 Hz and 20 Hz, sampled in 1 Hz increments. The corresponding time sampling was done in 50 ms increments for the duration of the trial, thus limiting the temporal resolution of the MSBF to 50 ms. The time-frequency sampling was done with a Gaussian filter, which resulted in broadening of each time-frequency point to a full power width at half maximum of ±1.42 Hz and ±78.8 ms. In processing of the time bins at epoch edges, BESA extends the epoch length in order to control for the possible artifacts that may result from filter edge effects. Following the complex demodulation method, linearly constrained minimum variance vector beamformer (e.g., [13]) was applied to each of the six 50 ms trial time bins in the frequency range between 4 and 8 Hz (theta band). The theta frequency band and the specific MSBF procedure described above were selected a priori based on previous work reported by Green and McDonald [11] that used MSBF to implicate theta band frequency (4–8 Hz) as a carrier frequency for volitional attentional control operations.

The location of the cortical sources was modeled using a standard head model. MSBF returned data depicting the change in theta band density (%q) for each 3×3×3 mm voxel relative to 50 ms pre-target baseline across participants. These values were subjected to nonparametric permutation tests [37], with the resulting significant group voxel values (p<.00001, uncorrected) displayed on the surface rendered three-dimensional brain images. It is important to note that the spatial resolution of these images is limited by the inherent constraints of EEG.

### Region of Interest (ROI) Analyses

The ROIs within MFG, TPJ, and IFG were defined based on the Talairach coordinates from the literature (TPJ [x = 52, y = 50, z = 28]; [38]; MFG [x = 45, y = 6, z = 46]; [7]; IFG [x = 48, y = 14, z = 10]; [9]). Each ROI subtended 9×9×9 mm area around the coordinate center. Mean theta density for each ROI across participants and time intervals was calculated from the raw data.
